# Binge Drinking and the Young Brain: A Mini Review of the Neurobiological Underpinnings of Alcohol-Induced Blackout

**DOI:** 10.3389/fpsyg.2018.00012

**Published:** 2018-01-19

**Authors:** Daniel F. Hermens, Jim Lagopoulos

**Affiliations:** ^1^Brain and Mind Centre, The University of Sydney, Sydney, NSW, Australia; ^2^Sunshine Coast Mind and Neuroscience Thompson Institute, University of the Sunshine Coast, Sunshine Coast, QLD, Australia

**Keywords:** binge drinking, alcohol-induced blackout, adolescent, young adult, hippocampus, memory, magnetic resonance spectroscopy

## Abstract

Binge drinking has significant effects on memory, particularly with regards to the transfer of information to long-term storage. Partial or complete blocking of memory formation is known as blackout. Youth represents a critical period in brain development that is particularly vulnerable to alcohol misuse. Animal models show that the adolescent brain is more vulnerable to the acute and chronic effects of alcohol compared with the adult brain. This mini-review addresses the neurobiological underpinnings of binge drinking and associated memory loss (blackout) in the adolescent and young adult period. Although the extent to which there are pre-existing versus alcohol-induced neurobiological changes remains unclear, it is likely that repetitive binge drinking in youth has detrimental effects on cognitive and social functioning. Given its role in learning and memory, the hippocampus is a critical region with neuroimaging research showing notable changes in this structure associated with alcohol misuse in young people. There is a great need for earlier identification of biological markers associated with alcohol-related brain damage. As a means to assess *in vivo* neurochemistry, magnetic resonance spectroscopy (MRS) has emerged as a particularly promising technique since changes in neurometabolites often precede gross structural changes. Thus, the current paper addresses how MRS biomarkers of neurotransmission (glutamate, GABA) and oxidative stress (indexed by depleted glutathione) in the hippocampal region of young binge drinkers may underlie propensity for blackouts and other memory impairments. MRS biomarkers may have particular utility in determining the acute versus longer-term effects of binge drinking in young people.

## Introduction

Binge drinking (BD) is the dominant type of alcohol misuse in young people (SAMHSA, 2009; Archie et al., 2012; Hermens et al., 2013). Alcohol use typically begins in adolescence with the prevalence of BD increasing sharply between 12 and 25 years old (to ∼40–50%), which is a pattern observed across Western countries (SAMHSA, 2011; Archie et al., 2012; AIHW, 2014; Schuckit et al., 2015). Although young people drink less frequently than older adults, they tend to drink more on each occasion (SAMHSA, 2009) and drinking to intoxication is especially common in teenagers (White and Hayman, 2006). Hence, single incident-excessive alcohol consumption or BD is often accompanied with adverse effects. These include increased risk of injury or accidental death, drink driving, unsafe sexual practices, periods of unconsciousness, as well as an increased likelihood of being a perpetrator or victim of assault (Bonomo et al., 2004; Mundt et al., 2012). A universal definition of BD remains lacking, however, it is generally accepted that it refers to “a single drinking session leading to intoxication” (Berridge et al., 2009). The USA’s National Institute on Alcohol Abuse and Alcoholism (NIAAA, 2017) has a more specific definition of: “a pattern of drinking that brings blood alcohol concentration (BAC) levels to 0.08 g/dL.” Furthermore, this would be within a period of about 2 h, which “typically occurs after four drinks for women and five drinks for men.” Despite this, numerous studies and surveys have opted for a simpler definition of BD as five or more drinks per single drinking occasion, for both sexes (SAMHSA, 2011; Degenhardt et al., 2013).

### Prevalence and Patterns of Binge Drinking in Young People

National surveys in the United States and Australia show that around 40% of young adults (aged ∼20–25 years^1^) report at least monthly BD. Similarly, in both countries around 5–6% of adolescents (aged 12–17 years) report this pattern of drinking (with a sharp increase to ∼15% by 16–17 years) (AIHW, 2017; SAMHSA, 2017). Across 35 European countries, around one third of 16 year olds report monthly BD (EMCDDA/ESPAD, 2016). The Australian survey (AIHW, 2017) also asked about any ‘loss of memory after drinking.’ Of those reporting monthly BD, 16–17 year olds had the highest rates of such memory loss (32%) with the next highest being the 20–24 year olds (24%). In terms of those with yearly but not monthly BD, 100% of 12–15 year olds reported alcohol-related memory loss, compared to the next highest group the 18–19 year olds (49%)^2^.

Longitudinal studies have provided important insights into the longer-term effects that adolescent BD may have on memory loss. Degenhardt et al. (2013) conducted a 15-year prospective study of *N* = 1943 Australians (from 14 to 15 years old) and found that 52% of males and 34% of females reported past-week adolescent BD. Furthermore, the vast majority continued to be BD into their adulthood and this was more likely in males, those who had antisocial behaviors and adverse consequences of drinking in adolescence. Notably, the adverse consequences included ‘intense drinking’ (i.e., when the subject could not remember the night before) as well as social problems, and alcohol-related sexual risk taking and injury/violence. Similarly, a longitudinal study of *N* = 1402 English adolescents who reported drinking alcohol prior to 15 years showed that 29% experienced alcohol-induced blackout (AIB)^3^. At follow-up, 57 and 74% had AIBs by 16 and 19 years, respectively (Schuckit et al., 2015). Although this study did not evaluate BD *per se*, the authors found that there was a general association between increased alcohol quantities and AIBs. One of the trajectories identified (30% of the sample) was thought to be prone to AIBs at age 16 due to links between their extroversion, peer substance use and BD (high BAC). However, the authors would not rule out other potential factors including family history of alcohol problems. Taken together, these findings suggest that young people who undertake BD are particularly prone to experiencing AIBs (Schuckit et al., 2015; Wetherill and Fromme, 2016). As a further complication, it remains a challenge to distinguish between the acute versus longer-term effects of BD in young people. These differential impacts of BD are addressed in the following sections.

### Early Binge Drinking: A Window of Vulnerability

The prevalence of BD in young people is particularly concerning given the damaging effects of alcohol on the developing adolescent-to-young adult brain (Hermens et al., 2013; Cservenka and Brumback, 2017). Despite this, there remains a relative paucity of neurobiological studies investigating the acute and longer-term effects of BD in young people (Hermens et al., 2013), particularly with respect to AIBs. Clark et al. (2008) suggest that the asynchronous development of the prefrontal cortex with respect to the limbic system in adolescence/young adulthood drives the heightened vulnerability to the effects of alcohol. Brain maturation continues well into the third decade of life, particularly in regards to prefrontal executive functions (EFs) (De Luca et al., 2003), which can result in an increased propensity for risky, impulsive behaviors and experimentation. In this period there are substantial changes in brain structure, with gray matter (GM) decreasing non-linearly in the cerebral cortex and linearly in the cerebellum and subcortical structures (caudate, putamen, pallidum), whereas in other subcortical structures (amygdala, hippocampus) slight, non-linear increases in GM volume are observed (Ostby et al., 2009). Additionally, white matter (WM) increases non-linearly in the cerebrum and cerebellum (Ostby et al., 2009). Hence, the period of adolescence-to-young adulthood is often viewed as a ‘window of vulnerability,’ particularly in the context of substance misuse (Bava and Tapert, 2010; Hermens et al., 2013). Young alcohol misusers first show impairments in memory and EF, which correspond with structural changes in hippocampal and prefrontal brain regions (Bava and Tapert, 2010; Hermens et al., 2013; Squeglia et al., 2015; Gropper et al., 2016; Wilson et al., 2017). Given its progressive development throughout adolescence the hippocampus is thought to be particularly susceptible to alcohol, including acute dysfunction causing blackout (Zeigler et al., 2005). Such dysfunction may be due to the increased sensitivity of the adolescent brain to the acute effects of alcohol and/or the maturational changes and associated heightened vulnerability driving longer-term effects of exposure. Due to ethics and legal issues, research on the acute effects of alcohol on younger people is not possible, and as such animal studies (see below) have been crucial in our understandings of how the adolescent brain is particularly vulnerable to BD (Zeigler et al., 2005). Despite this, several human studies have provided important insights into the cognitive effects of acute alcohol ingestion. Acheson et al. (1998) conducted a randomized, repeated-measures placebo-controlled trial of alcohol (0.6 g/kg) in *N* = 12 healthy adults. They found that compared to placebo alcohol significantly impaired the acquisition of both semantic and non-verbal memory. Importantly, younger subjects (21–24 years) performed worse in the alcohol condition compared to their older peers (25–29 years) in immediate and delayed recall (visuo-spatial) and delayed recognition (verbal memory). Similarly, Vinader-Caerols et al. (2017) examined the acute effects of alcohol (i.e., doses of 0, 0.3–0.5, or 0.54–1.1 g/L) in past 12-month refrainers or BD aged 18–19 years. Compared to their BD and non-drinking peers those who consumed the highest acute dose showed the most impaired immediate visual and working memory, while the lower dose BD group showed impaired immediate visual memory only.

Other studies have examined the potential longer-term, dose-dependent effects of BD on cognitive performance. Nguyen-Louie et al. (2016) examined verbal learning and memory in adolescents (12–16 years) who were determined (6 years after baseline) to be moderate, binge or extreme-binge drinkers (≤4, 5+, or 10+ drinks/occasion). At follow-up, the extreme-BD group performed significantly worse than the moderate drinkers in verbal learning, as well as cued and free short delayed recall (BD performed at an intermediate level). Furthermore, for every additional drink consumed in adolescence, there was a linearly increasing deleterious effect on a range of learning, recall and recognition measures. In contrast, a more recent longitudinal study (Boelema et al., 2015) of *N* = 2230 Dutch adolescents found no differences among non-, light-, and heavy-drinkers in terms of the maturation of four measures of EF (i.e., inhibition, working memory, and shift- and sustained attention).

### Animal Models

Earlier studies by Swartzwelder and colleagues utilized rat hippocampal slices to demonstrate the effects of acute alcohol exposure on the pre-pubertal/adolescent brain. Swartzwelder et al. (1995b) showed that alcohol has greater suppression of *N*-methyl-D-aspartate (NMDA) receptor-mediated synaptic potentials in pre-pubertal as compared with adult rats. Thus, the authors suggested that young drinkers may be at greatest risk of compromised cognitive function (i.e., anterograde memory formation) related to hippocampal NMDA activity. In other similar studies, this group provided further evidence of perturbed hippocampal function in adolescent but not adult rats; with attenuated long-term potentiation (LTP; important in the acquisition of spatial memory as well as learning and memory formation or ‘synaptic plasticity’) being observed across three different doses, including those more representative of human intoxication (Swartzwelder et al., 1995a; Pyapali et al., 1999). More recently, Risher et al. (2015) utilized ‘adolescent intermittent ethanol’ exposure via intragastric gavage for 16 days (until adulthood) before examining the acute effects of alcohol on hippocampal slices, and found enduring structural and functional abnormalities, reflecting synaptic immaturity.

Two subsequent studies probed and evaluated the longer-term effects of alcohol in adolescent and adult rats performing memory tasks. Markwiese et al. (1998) injected rats with alcohol (1.0 or 2.0 g/kg) or saline 30 min before trials on a spatial memory task, over a 5-day period. Notably, alcohol significantly impaired adolescent but not adult rats in spatial memory acquisition. As a follow-up to this, White et al. (2000) exposed rats to binge-style alcohol (i.e., 5.0 g/kg, 48-h intervals) or saline over a 20 day period. Animals were then tested (20 days post final dose) on an elevated plus maze and trained to perform spatial working memory task. Interestingly, prior exposure to alcohol and group status did not affect plus maze behavior nor spatial working memory performance, however, the animals exposed to binge-style alcohol as adolescents showed significant impairments in working memory when undertaken during an alcohol challenge (1.5 g/kg) compared to the other three groups (including binge-exposed adults). Importantly, the overall findings of studies utilizing intraperitoneal injections have been observed in similar studies utilizing self-administration protocols. Vargas et al. (2014) showed that voluntary binge drinking during adolescence produced enduring WM deficits in prefrontal circuitry and poorer performance in working memory, which was over and above the effects of vapor exposure (modeling dependence; over a longer period) during adulthood, suggesting that the adolescent brain has a heightened sensitivity to alcohol.

### Acute Alcohol Use, Memory Loss: Blackout

‘Blackout’ or the loss of memory during an episode of drinking was first documented as an important indicator of alcoholism (Jellinek, 1946). However, it is now understood as phenomenon that can be experienced by any drinker, as it is typically induced by BD with a rapid increase in BAC; although there are a range of factors that are thought to increase the likelihood of blackout (Rose and Grant, 2010). Most definitions of blackout refer to there being a breakdown in the transfer of information from short-term to long-term storage (Acheson et al., 1998; White, 2003; Siqueira and Smith, 2015). Importantly, this occurs while immediate (very brief short-term) and remote (long-term; formed prior to intoxication) memory abilities remains intact (White, 2003). More specifically, an AIB leads to a failure in forming new explicit memories (i.e., facts and events) (Lister et al., 1991). Such anterograde amnesia occurs despite the subject continuing to participate in events (e.g., holding a conversation) that they will not remember later (White, 2003; Lee et al., 2009).

There is no objective test to determine that one is experiencing a blackout (Goodwin, 1995; Pressman and Caudill, 2013; Wetherill and Fromme, 2016). Thus, observers rely on the subject’s self-report which is itself constrained by the concept of being asked to ‘remember not remembering’ (Wetherill and Fromme, 2016). Detailed research has led to the identification of two qualitatively different types of blackouts: ‘en bloc’ (complete) and fragmentary (partial), first described almost 50 years (Goodwin et al., 1969a,b) these terms remain valid today (White, 2003; Rose and Grant, 2010). AIBs should not be confused with losing consciousness (i.e., “passing out”), rather an AIB is the memory lost from the conscious state whereby en bloc blackouts represent the complete interruption of memory transfer (an absence of encoding) and fragmentary blackouts (FBs) reflect partial obstruction of memory formation (a deficiency of encoding), which may be ameliorated via cueing (Lee et al., 2009; Rose and Grant, 2010).

For Acheson et al. (1998), AIBs stems from two processes: first, alcohol reduces one’s ability to process new information (Maylor and Rabbitt, 1993), then it facilitates faster forgetting (Maylor and Rabbitt, 1987). Importantly, rapid forgetting is a hallmark of hippocampal dysfunction (Squire et al., 2004), however, not all BD experience blackout, implying that genetic factors also play a role (Lee et al., 2009). Genetic epidemiological research supports this assumption. An Australian study of 2324 twin pairs reported a 52.5% heritability rate of lifetime AIBs (Nelson et al., 2004). Interestingly, it was speculated that genes whose products mediate alcohol’s effects on hippocampal neurotransmission probably underlie such risk. On the other hand, early alcohol exposure may have specific impacts on longer-term hippocampal functioning as suggested by a longitudinal study of *N* = 1145 young adults (Marino and Fromme, 2016). Whereby, earlier drinking age was associated with more frequent blackouts (over 3-year period) which persisted despite a reduction in BD episodes.

A paucity of neuroimaging studies has directly examined AIB. However, functional magnetic resonance imaging (fMRI) studies undertaken to date provide evidence for neurobiological vulnerabilities that may exist prior to alcohol use onset and become more evident after BD patterns emerge (Wetherill and Fromme, 2016). Wetherill et al. (2012) utilized two fMRI sessions (nil vs. alcohol ingestion) to compare *N* = 12 university students (21–23 years) with a past 12-month history of FB to *N* = 12 peers without FB in a contextual memory task. The groups did not differ in performance or neural activity during the nil alcohol session. However, in the alcohol session (0.08% breath alcohol concentration) the FB group showed decreased blood-oxygen-level dependency (BOLD) response during encoding and recollection of contextual details in dorsolateral prefrontal and parietal regions.

Subsequently, this same group conducted an fMRI study in substance-naïve 13 year olds (Wetherill et al., 2013). At 5-year follow-up, the investigators compared inhibitory processing in those who remained substance naïve (*n* = 20) versus those who had transitioned into heavy drinkers with (*n* = 20) or without (*n* = 20) a history of AIB. Interestingly, at baseline the AIB group showed greater activation (increased BOLD) in frontal and cerebellar brain regions during inhibitory processing compared to both other groups. The authors suggested this provided evidence of inherent vulnerabilities to inhibitory processing difficulties that likely contribute to alcohol-induced memory impairments (Wetherill and Fromme, 2016).

### Magnetic Resonance Spectroscopy: Probing the Neurochemistry of Blackout

Magnetic resonance spectroscopy (MRS) has provided evidence of *in vivo* neurochemical perturbations associated with alcohol misuse in human (Lee et al., 2007; Hermann et al., 2012; Ende et al., 2013; Yeo et al., 2013) and animal (Hermann et al., 2012) studies. However, only two MRS studies have specifically examined AIBs. Silveri et al. (2014) examined neurochemical profiles in the frontal and parietal-occipital lobes of BD aged 18–24 years. Compared to their light-drinking (LD) peers (*N* = 31), BD (*N* = 21) showed reduced gamma-aminobutyric acid (GABA) and *N*-acetylaspartate (NAA; a marker of neuronal integrity) in the anterior cingulate cortex (ACC). Furthermore, BD with a history of AIBs also showed significantly reduced glutamate compared LD. Follow-up analyses suggested that the reductions in GABA and NAA were more pronounced in BD with AIBs. There was also a trend for a reduction in glutamate in this subgroup. Importantly, all subjects had experience as college students, had high-average to superior IQ and none had an alcohol use disorder (AUD). Thus, the authors suggested that these findings might serve as early markers of risk in young individuals who continue hazardous drinking. Notably, only GABA was found to be significantly associated with cognitive performance, with lower levels of ACC-GABA being associated with worse performance in attentional switching and response inhibition.

To our knowledge, only one other study has specifically investigated AIB utilizing MRS. Our group (Chitty et al., 2014) examined the relationship between *in vivo* glutathione (GSH; the brain’s primary anti-oxidant) levels in young people with bipolar disorder (aged 18–30 years), given the high levels of alcohol use common to this psychiatric group and alcohol’s propensity to trigger oxidative stress (via the production of reactive oxygen species) in the brain (Nordmann et al., 1990). Despite no significant difference in overall risky drinking levels compared to healthy controls, the bipolar disorder group showed an association between increased alcohol use and decreased frontal (ACC) and hippocampal GSH. We supposed that this association might be evidence of memory impairment related to alcohol-induced oxidation, since increases in oxidative stress have also been linked to impairments in synaptic plasticity and memory, and decreased capacity to exhibit LTP (Pellmar et al., 1991; Auerbach and Segal, 1997).

### Hippocampus: The Target of Further Investigation

Although mechanisms around AIBs are becoming increasingly understood, a detailed understanding of the neurobiological vulnerability (and why some individuals experience blackouts) remains unknown (Wetherill and Fromme, 2016). We would argue that more research targeting the neurochemistry and functioning of the hippocampus is needed to address this. More broadly, the hippocampus has been implicated in the pathogenesis of AUD (White and Swartzwelder, 2004). Furthermore, a substantive amount of work has led to the hippocampus being a focal point in studies of both the acute and chronic effects of alcohol use (Abrahao et al., 2017), particularly given its inhibition of glutamate binding [suppression of NMDA receptors (NMDAr)] (Strelnikov, 2007). It is also well-established that with chronic alcohol use, NMDAr binding sites increase in number and level of functioning (up-regulation), as demonstrated in rodents who show increased glutamate transmission in the hippocampus after repeated ethanol administration (Chefer et al., 2011). Furthermore, upon alcohol withdrawal, excessive glutamate activity resulting from increased numbers of NMDAr leads to a state of excitoxicity that can contribute to neurodegeneration (Hunt, 1993). Thus, periods of BD followed by abstinence may trigger cycles of neural responses that facilitate such neurotoxicity and associated cognitive impairments (Zeigler et al., 2005). Future studies should explore this by specifically examining factors associated with (and without) AIB, in particular, the underlying neurochemistry. This is crucial given the two key mechanisms underlying AIBs (Rose and Grant, 2010); that is: (i) a breakdown or blocking of short-term memory transfer, followed by; (ii) compromised subsequent retrieval caused by disruptions in hippocampal pyramidal cell activity. Crucially, the neurochemical processes underpinning these steps are: (i) potentiation of GABA-mediated inhibition; and (ii) interference of hippocampal NMDAr activation, leading to decreased LTP (Rose and Grant, 2010). The role of GSH may be important too given its status as a marker of oxidative stress. Furthermore, glutamate is a precursor of both GABA and GSH therefore the relationship between these metabolites (all measured via MRS) may be crucial to understanding individual differences in AIBs.

## Author Contributions

All authors listed have made a substantial, direct and intellectual contribution to the work, and approved it for publication.

## Conflict of Interest Statement

The authors declare that the research was conducted in the absence of any commercial or financial relationships that could be construed as a potential conflict of interest.

## Footnotes

1 The age range in the Australian Institute of Health Welfare (AIHW) survey was 20–24 years; whereas in the Substance Abuse and Mental Health Services Administration (SAMHSA) survey it was 20–25 years.

2 For the 16–17 year old group with yearly but not monthly BD, the rate of ‘loss of memory after drinking’ could not be confirmed because of high sampling error.

3 Schuckit et al. (2015) used the term ‘alcohol-related blackout’ however, alcohol-induced blackout is more commonly used and therefore “AIB” is term used throughout this paper.
